# Construction of a circRNA-miRNA-mRNA network based on differentially co-expressed circular RNA in gastric cancer tissue and plasma by bioinformatics analysis

**DOI:** 10.1186/s12957-022-02503-7

**Published:** 2022-02-14

**Authors:** Yu Gong, Yuwen Jiao, Xiaoyang Qi, Jinjin Fu, Jun Qian, Jie Zhu, Haojun Yang, Liming Tang

**Affiliations:** 1 Department of Gastroenterology Surgery, The Affiliated Changzhou No.2 People’s Hospital of NanJing Medical University, Changzhou, Jiangsu China; 2 Department of Gastroenterology, The Affiliated Changzhou No.2 People’s Hospital of NanJing Medical University, Changzhou, Jiangsu China

**Keywords:** circRNA, MicroRNA sponge, Gastric cancer, GEO

## Abstract

**Background:**

Increasing evidence implicates circular RNAs (circRNAs) have been involved in human cancer progression. However, the mechanism remains unclear. In this study, we identified novel circRNAs related to gastric cancer and constructed a circRNA-miRNA-mRNA network.

**Methods:**

Microarray datasets GSE83521 and GSE93541 were obtained from the Gene Expression Omnibus (GEO). Then, we used computational biology to identify circRNAs that were differentially expressed in both GC tissue and plasma compared to normal controls; then, we detected the expression of the selected circRNAs in gastric cell lines by quantitative real-time polymerase chain reaction (qRT-PCR). We also identified circRNA-related candidate miRNAs and their target genes with online tools. Combining the predicted miRNAs and target mRNAs, a competing endogenous RNA regulatory network was established. Functional and pathway enrichment analyses were performed, and interactions between proteins were predicted by using String and Cytoscape. Gene ontology (GO) and Kyoto Encyclopedia of Genes and Genomes (KEGG) analyses were performed to elucidate the possible functions of these differentially expressed circRNAs. The regulatory network constructed using the microarray datasets (GSE83521 and GSE93541) contained three differentially co-expressed circRNAs (DECs). A circRNA-miRNA-mRNA network was constructed based on 3 circRNAs, 43 miRNAs and 119 mRNAs.

**Results:**

GO and KEGG analysis showed that the regulation of apoptotic signaling pathway and PI3K−Akt signaling pathway were highest degrees of enrichment respectively. We established a protein-protein interaction (PPI) network consisting of 165 nodes and 170 edges and identified hub genes by using MCODE plugin in Cytoscape. Furthermore, a core circRNA-miRNA-mRNA network was constructed based on hub genes. Hsa_circ_0001013 was finally determined to play an important role in the pathogenesis of GC according to the core circRNA-miRNA-mRNA network.

**Conclusions:**

We propose a new circRNA-miRNA-mRNA network that is associated with the pathogenesis of GC. The network may become a new molecular biomarker and could be used to develop potential therapeutic strategies for gastric cancer.

## Introduction

Gastric cancer is one of the most common malignant tumors. Every year, there are nearly 1 million new cases of gastric cancer around the world, making it the third leading cause of cancer-related deaths and prompting the World Health Organization to declare it a public health problem [1]. The pathogenesis of gastric cancer is multifactorial and involves multiple steps, but these steps are currently unclear. It is widely believed that *Helicobacter pylori* is one of the main pathogenic factors of gastric cancer [2, 3]. Almost 90% of new cases of non-cardiac gastric cancer are related to *Helicobacter pylori*. Although the techniques for the detection and treatment of gastric cancer have dramatically improved, the prognosis is still very poor [4]. The 5-year survival rate of patients with advanced gastric cancer is approximately 18–29% [5]. Therefore, early diagnosis and treatment are critical to improving the curative effect and reducing the mortality rate.

In 1976, a new type of 3′-5′ head-to-tail covalently closed RNA called circular RNAs (circRNAs) were identified [6, 7]. However, in subsequent decades, circular RNAs were thought to be the product of mis-splicing [8]. In recent years, it has been recognized that circRNAs are normal co-products of numerous eukaryotic protein-coding genes [9]. CircRNAs have been a hotspot of research in the fields of life science and medicine and have been identified as critical regulators of a variety of diseases, including various malignant tumors [10–12]. CircRNAs can regulate variable splicing or the expression of their host genes by blocking transcriptional initiation sites, and can even be translated into proteins or peptides. However, the role of competitive endogenous RNA (ceRNA) in sponging miRNA is considered to be one of the main functions of circRNA in various cancers. CircRNA is also regarded as a potential biomarker for cancers because it is more stable than linear RNA [13]. There are many differentially expressed circRNAs associated with gastric cancer. For example, Wei et al. found that circHIPK3 promotes the proliferation and migration of gastric cancer cells by sponging miR-107 and regulating BDNF expression [14, 15]. He et al. confirmed that circular RNA circ_0006282 contributes to the progression of gastric cancer by sponging miR-155 to upregulate the expression of FBXO22 [16]. Pan et al. reported that circUBA1 promotes gastric cancer proliferation and metastasis by acting as a competitive endogenous RNA by sponging miR-375 and regulating TEAD4 [17]. Additionally, circRNAs have also been proposed as diagnostic or prognostic biomarkers. Wang et al. demonstrated that hsa_circ_0005654 might serve as a new and promising diagnostic biomarker for early gastric cancer screening. The AUC, sensitivity, and specificity of hsa_circ_0005654 are significantly higher than those of present gastric cancer associated-biomarkers [18]. Although related studies have emerged, the network involved in circRNA-mediated regulation of gastric cancer remains unclear.

**Fig. 1:**
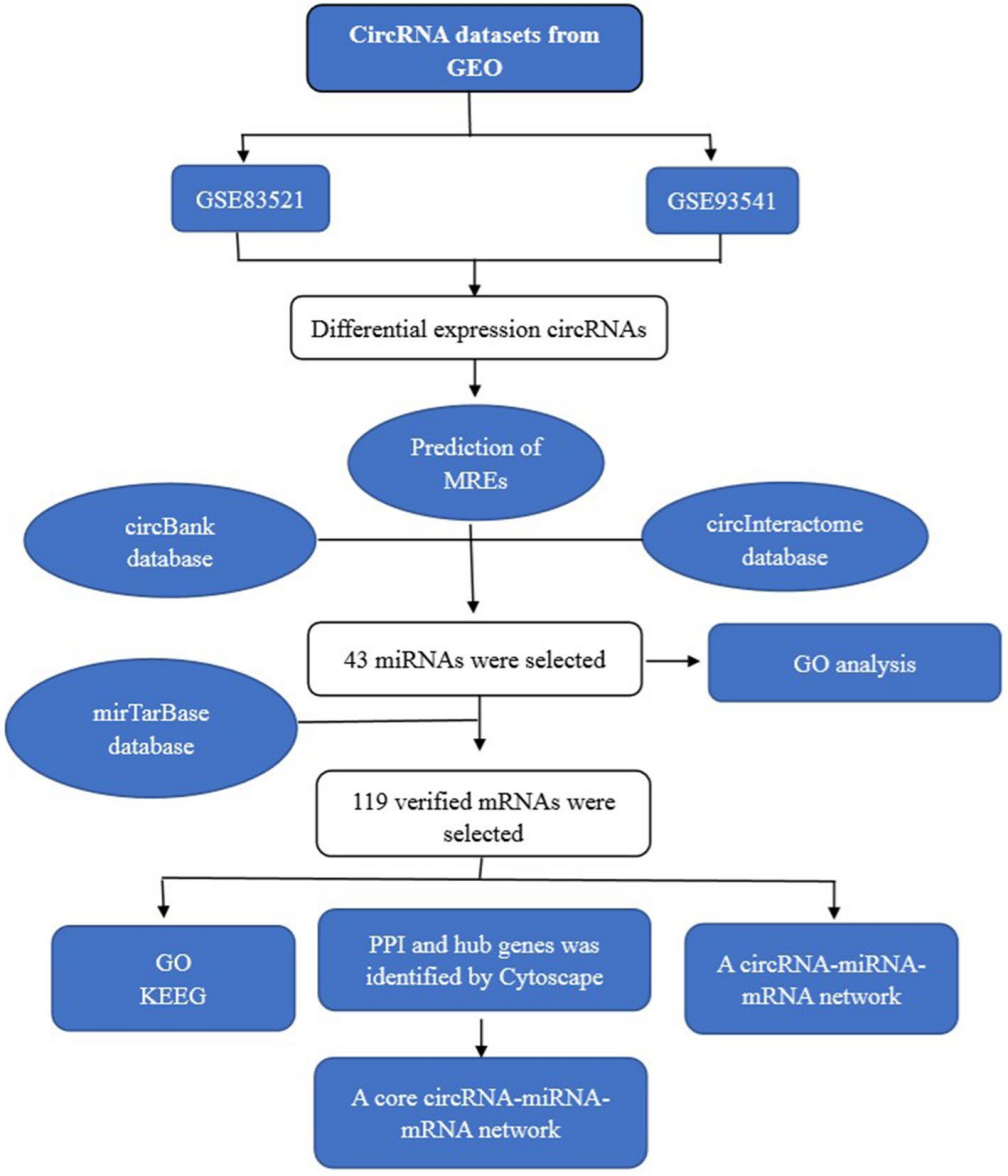
Flow chart of the methods used in the present study. *GO* gene ontology, *circRNA* circular RNA, *KEGG* Kyoto Encyclopedia of Genes and Genome, *mRNA* messenger RNA, *PPI* protein-protein interaction

The aim of our study was to identify differentially co-expressed circRNAs in tissues and plasma of patients with gastric cancer. The expression profiles of circRNAs were obtained from the Gene Expression Omnibus (GEO). The bioinformatic data were analyzed and differentially expressed circRNAs (DECs) were screened. Next, the potential miRNAs sponged by DECs and their target genes were identified by bioinformatic analysis. Moreover, the core circRNA-miRNA-mRNA regulatory network was constructed. Gene enrichment analyses of the candidate miRNAs or mRNAs were performed with the gene ontology (GO) and Kyoto Encyclopedia of Genes and Genomes (KEGG) databases and R software, which resulted in the prediction of the signaling pathways involved in GC. A flow chart of the methods used in this study is provided in Fig. 1. The results of this study may help to clarify the potential mechanism of the pathogenesis of gastric cancer, provide new biomarkers for gastric cancer and facilitate future research in GC treatment and diagnosis.

## Methods

### Microarray analysis of gene expression

Two circRNA expression profiles for human samples derived from patients with gastric cancer were obtained from GEO (www.ncbi.nlm.nih.gov/geo/). We chose the GSE83521 and GSE93541 circRNA expression profiles, both of which were generated from the Agilent-069978 Arraystar Human CircRNA microarray V1 GPL19978 platform. The GSE83521 dataset contained six gastric cancer tissues and six normal mucosa tissues, and the GSE93541 dataset included three plasma samples of gastric cancer patients and three healthy controls.

### Identification of DECs

Differential expression of the circRNAs in the two datasets was analyzed by using the GEO2R online analysis tool. The absolute value of log fold change > 1.5 and *p* value < 0.05 were used as cut-off criteria. The significantly differentially expressed circRNAs in the two datasets were screened, and the co-expressed circRNAs were detected by Venn analysis. The basic structural features of the differentially expressed circRNAs were obtained from the Cancer-Specific CircRNA Database (http://gb.whu.edu.cn/CSCD/).

### Prediction of circRNA-miRNA and miRNA-mRNA interactions

Online tools circBank (http://www.circbank.cn/) and Circular RNA Interactome (https://circinteractome.nia.nih.gov/index.html) were used to predict the possible interactions between circRNAs and miRNAs. The miRNAs predicted by both circBank and circInteractome were selected as candidate miRNAs. mirTarBase (http.//mirtarba se.mbc.nctu.edu.tw/php/index.php) was used to obtain experimentally strongly supported target genes of these miRNAs. Only candidate genes that could be verified by reporter assays as well as Western blot or qPCR experiments were selected.

### GO and KEGG functional enrichment analysis

FunRich software (version 3.1.1) was used to conduct GO analysis for candidate miRNAs. GO annotation and KEGG pathway analyses were conducted with the R (version 3.6) package (http://www.bioconductor.org/) clusterProfiler to explore the potential biological roles of candidate genes. The analysis results were visualized with the ggplot2 package of R software. Both a *p* value and *q* value < 0.05 were considered significant for GO annotation, while a *p* value < 0.05 and *q* value < 1 were considered significant for KEGG pathway analysis.

### Construction of the protein-protein interaction and circRNA-miRNA-mRNA network

Candidate target genes of the candidate miRNAs were put into the Search Tool for the Retrieval of Interacting Genes database (STRING, https://string-db.org/), and an interaction network chart with a combined score > 0.4 was saved and exported. Then the protein-protein interaction (PPI) network was visualized using Cytoscape software (version 3.6.1; http://cytoscape.org/). The MCODE plugin of Cytoscape software was used to identify hub genes among candidate targets. The circRNA–miRNA–mRNA network was also visualized by Cytoscape software.

### Cell culture

The gastric cancer cell line SGC-7901 and human gastric mucosal epithelial cell line GES-1 were purchased from the Cell Resource Center, Shanghai Institute of Biochemistry and Cell Biology, Chinese Academy of Science. All of the cell lines were maintained under the recommended culture conditions and incubated at 37 °C in a humidified environment with 5% CO_2_.

### RNA extraction and quantitative real-time polymerase chain reaction

Total RNA was isolated from the cell lines with TRIzol reagent (Invitrogen, CA, USA) following the manufacturer’s instructions. The concentration and purity of the total RNA samples were assessed using a NanoDrop spectrophotometer (Thermo, Wilmington, DE, USA). Total RNA was reverse transcribed using HiScript Q RT SuperMix for qPCR with gDNA wiper (Vazyme Biotech, Nanjing, China), and qPCR assays were performed in triplicate using the AceQ qPCR SYBR Green Master Mix kit (Vazyme Biotech, Nanjing, China) on a 7500 real-time PCR system (ABI). The divergent primers used for detecting circRNAs were synthesized from Shanghai Generay Biotech (Shanghai, China), and β-actin was used as an internal control. The following primer pairs were used for qPCR: β-actin forward, 5′-AGAAAATCTGGCACCACACC-3′ and reverse, 5′-CAGAGGCGTACAGGGATAGC-3′; hsa_circ_0001013 forward, 5′-GTCAAAGGAAGCAAAAGAAAGTCT-3′ and reverse, 5′-GATCGCACCTCTACACTCCA-3′; hsa_circ_0007376 forward, 5′-ATCGACTCCATGGCCAACTC-3′ and reverse, 5′-AAGCCCCGGAGAACAGC-3′; hsa_circ_0043947 forward, 5′-CAATTGTGGTTGTGCAGCC-3′ and reverse, 5′-ACACAAACTCAGCATCATGGA-3′. The expression of circRNAs was normalized to that of the internal control β-actin by using the 2^-ΔΔC^ method [19].

### Statistical analysis

All computations were carried out using GraphPad Prism 8 (GraphPad Software, CA, USA). Data are expressed as mean ± SEM. Student’s *t* test was conducted to compare the differences in circRNA expression between GES-1 and SGC-7901 cells. *P* < 0.05 was considered statistically significant.

## Results

### Identification of DECs

**Fig. 2:**
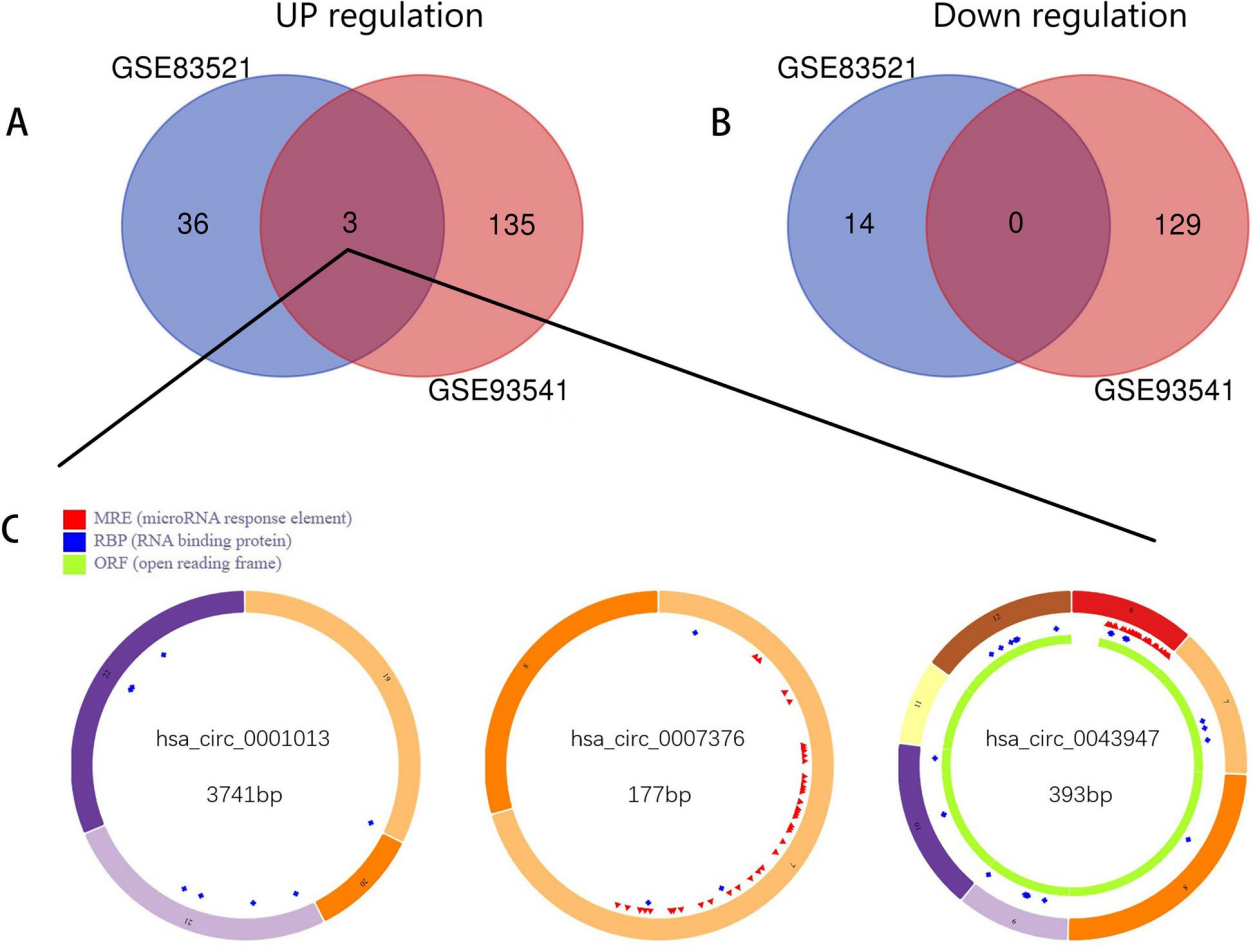
Differentially co-expressed circRNAs in gastric cancer tissues and plasma. **A**, **B** Venn diagram used to select the three overlapping differentially expressed circRNAs detected by analysis of the GSE89143 and GSE93541 datasets. **C** The essential characteristics and basic structural patterns of DECs were analyzed by the cancer-specific circRNA database. *MRE* miRNA response element, *RBP* RNA binding protein, *ORF* open reading frame

Two circRNA expression profiles, GSE83521 and GSE93541, were obtained from GEO, and the GEO2R method was applied to analyze DECs. The GSE83521 dataset was derived from gastric cancer tissues, and the GSE93541 dataset was derived from plasma samples. CircRNAs that were differentially expressed both in the tissues and the plasmas of gastric cancer patients were selected as our target circRNAs. We found that 53 circRNAs were differentially expressed in GSE83521, including 39 upregulated and 14 downregulated circRNAs, while 267 differentially expressed circRNAs were identified in GSE93541, including 138 upregulated and 129 downregulated circRNAs. Among them, 3 common upregulated and 0 common downregulated circRNAs were observed in the two circRNA expression profiles. A Venn diagram of the results is shown in Fig. 2A and B. The upregulated circRNAs that overlapped in the two datasets (hsa_circ_0001013, hsa_circ_0007376, hsa_circ_0043947) were selected for further analysis. Details of the overlapping upregulated circRNAs are listed in Table 1, and the basic structural features of the three selected circRNAs are shown in Fig. 2C.

**Table 1:** Features of 3 selected circRNAs

CircRNA ID	GSE83521		GSE93541		Chromosome location	Gene symbol	Accession number
	*P* value	Log2FC	*P* value	Log2FC			
hsa_circ_0001013	0.0003	1.9539	0.0023	1.7488	Chr2:61339656-61345251+	KIAA1841	NM_001129993
hsa_circ_0007376	0.0000	1.9983	0.0199	3.2983	Chr19:4101016-4101278-	MAP2K2	NM_030662
hsa_circ_0043947	0.0289	1.8146	0.0000	3.1526	Chr17:41199659-41215968-	BRCA1	NM_007300

### Expression of circRNAs in datasets and cell lines

**Fig. 3:**
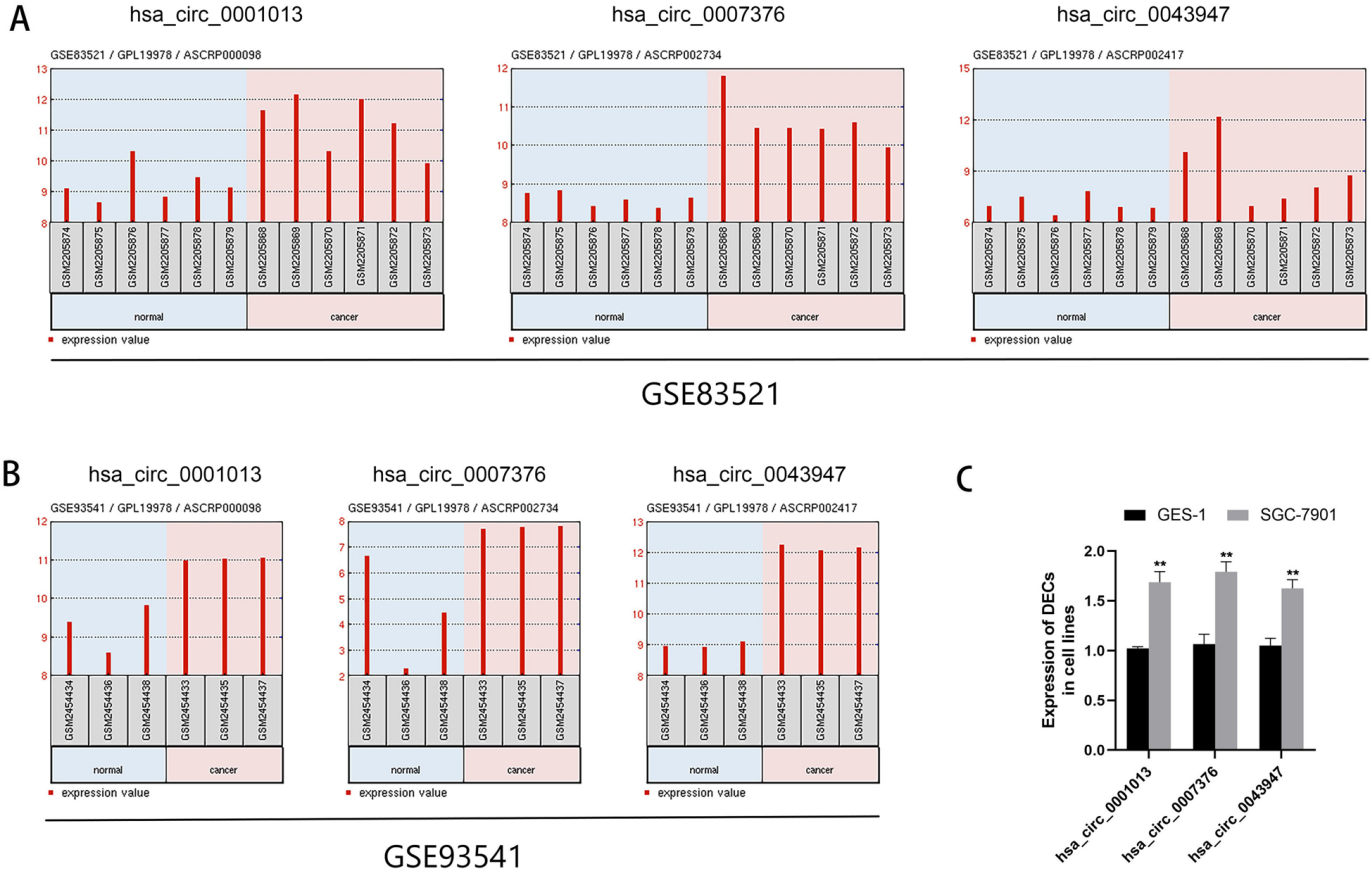
Expression of DECs in gastric cancer samples and cell lines. **A** The expression of three selected circRNAs in six gastric cancer tissues and six normal mucosa tissues. **B** The expression of three selected circRNAs in three gastric cancer plasmas and three healthy controls. **C** The relative expression of three selected circRNAs in gastric cancer cell line SGC-7901 and human gastric epithelial cell line GES-1. **< 0.05

As shown in Fig. 3A and B, the expression patterns of the three selected circRNAs were upregulated in both tissues and plasma samples according to the datasets. We also detected the expression of selected circRNAs in the gastric cancer cell line SGC-7901 and the human gastric epithelial cell line GES-1 by qRT-PCR. The results showed that all three selected circRNAs had higher expression levels in SGC-7901 than in GES-1 as shown in Fig. 3C.

### Prediction of circRNA-miRNA and their functional analysis

**Fig. 4:**
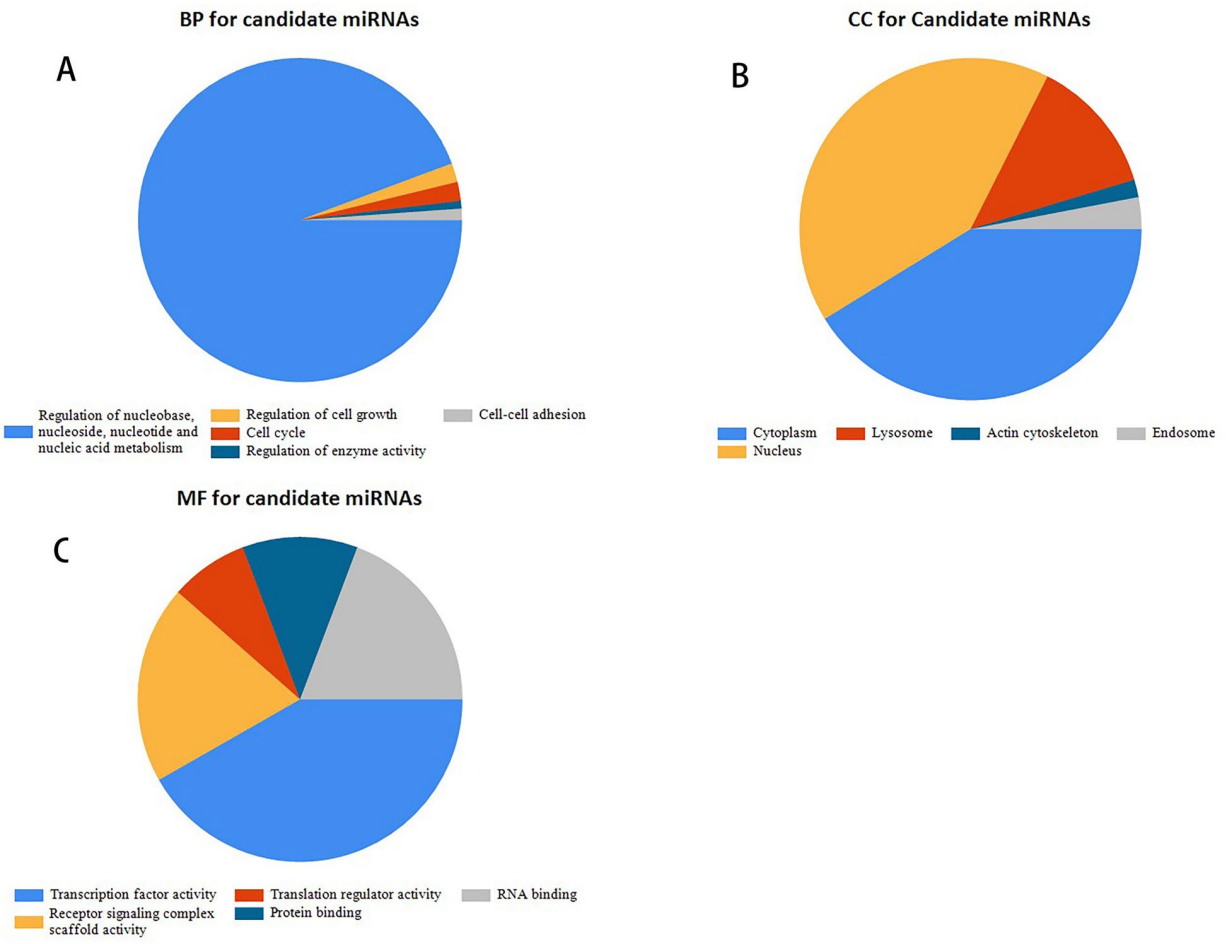
GO analysis for 43 miRNAs by RichFun software. **A**–**C** Top five enrichment items for BP, CC, and MF respectively. *BP* biological progress, *CC* cellular component, *MF* molecular function

An increasing amount of evidence demonstrates that circRNAs might function as competing endogenous RNAs (ceRNAs) that operate by competitively binding common microRNAs (miRNAs) and increase the expression of the target genes of these miRNAs. Target miRNAs of the three selected circRNAs were predicted by two online tools, circBank and circInteractome. A total of 43 consensus miRNAs from both prediction tools were identified and DECs potentially binding to these miRNAs are presented in Table 2. The results showed that one specific circRNA might bind to more miRNAs, while different circRNAs could interact with one specific miRNA. FunRich software was used for GO analysis of the 43 miRNAs. The top five enriched terms are shown in Fig. 4: “regulation of nucleobase, nucleoside, nucleotide and nucleic acid metabolism,” “regulation of cell growth,” “Cell cycle,” “regulation of enzyme activity”: and “cell-cell adhesion’ for biological progress (BP),” “cytoplasm and nucleus,” “nucleus,” “lysosome,” “actin cytoskeleton” and “endosome” for cellular component (CC), and “transcription factor activity,” “receptor signaling complex scaffold activity”, “translation regulator activity”, “protein binding” and “RNA binding” for molecular function (MF). All of these results indicated that circRNAs might impact on GC progression by modulating various miRNAs.

**Table 2:** Selected circRNAs, miRNAs interact with circRNAs and their target genes

	miRNA	mRNA
hsa_circ_0001013	hsa-miR-1197, hsa-miR-1225-3p, hsa-miR-1243, hsa-miR-1250-5p, hsa-miR-1261, hsa-miR-1294, hsa-miR-1304-5p, hsa-miR-146b-3p, hsa-miR-1827, hsa-miR-323a-3p, hsa-miR-450b-3p, hsa-miR-548g-3p, hsa-miR-548m, hsa-miR-556-3p, hsa-miR-562, hsa-miR-576-5p, hsa-miR-624-3p, hsa-miR-924	None
	hsa-miR-1228-3p	CSNK2A2, TP53
	hsa-miR-1256	TRIM68
	hsa-miR-1283	ATF4
	hsa-miR-136-5p	MTDH, PPP2R2A, RASAL2, IL6
	hsa-miR-182-5p	CDKN1A, FOXO3, FOXO1, RARG, MITF, ADCY6, CLOCK, TSC22D3, FGF9, NTM, CYLD, BCL2, CCND2, PDCD4, RECK, FLOT1, PTEN, GSK38, ZFAND4, BDNF, SATB2, CHL1, CADM1, TP53INP1, TCEAL7, FBXW7, LRRC4, ULBP2, PDK4, TRIM8, TIAM1, UQCRFS1
	hsa-miR-197-3p	FOXO3, TUSC2, NSUN5, CD82, BMF, PMAIP1, MTHFD1, FOXJ2, MAPK1
	hsa-miR-330-5p	MUC1, ITGA5, PDE4B
	hsa-miR-337-3p	RAP1A, STAT3, CSNK2A1, MZF1
	hsa-miR-451a	MIF, CAB39, ABCB1, MYC, RAB14, CPNE3, RAB5A, DCBLD2, IL6R, ADAM10, TSC1, MAPK1, CDKN2D, MAP3K1, IL6
	hsa-miR-487a-3p	ABCG2, SPRED2, PIK3R1
	hsa-miR-488-3p	SLC39A8, PAX6, BCL2L11
	hsa-miR-510-5p	SPDEF, PRDX1,
	hsa-miR-513a-3p	GSTP1, LHCGR
	hsa-miR-548c-3p	ITGAV, TWIST1
	hsa-miR-570-3p	CD274
	hsa-miR-654-3p	CDKN1A
	hsa-miR-665	CD274, CNR2
	hsa-miR-876-3p	MCL1
	hsa-miR-942-5p	CDKN1A, IFI27, SFRP4, GSK3B, TLE1, NFKBIA
hsa_circ_0007376	hsa-miR-571	None
	hsa-miR-224-5p	KLK10, CXCR4, CDC42, API5, EYA4, EDNRA, DIO1, SMAD4, PEBP1, TCEAL1, PHLPP1, HOXD10, PTX3, MBD2, TPD52, TRIB1, CDH1, APLN, CASP7, CASP3, MTOR, PHLPP2, RASSF8
hsa_circ_0043947	hsa-miR-1257	None
	hsa-miR-140-3p	NRIP1, CD38, ATP6AP2, ITGA6, MARCKSL1, COL4A1, ATP8A1
	hsa-miR-151a-3p	TWIST1, IL12RB2
	hsa-miR-660-5p	TFCP2

### Construction of the ceRNA network

**Fig. 5:**
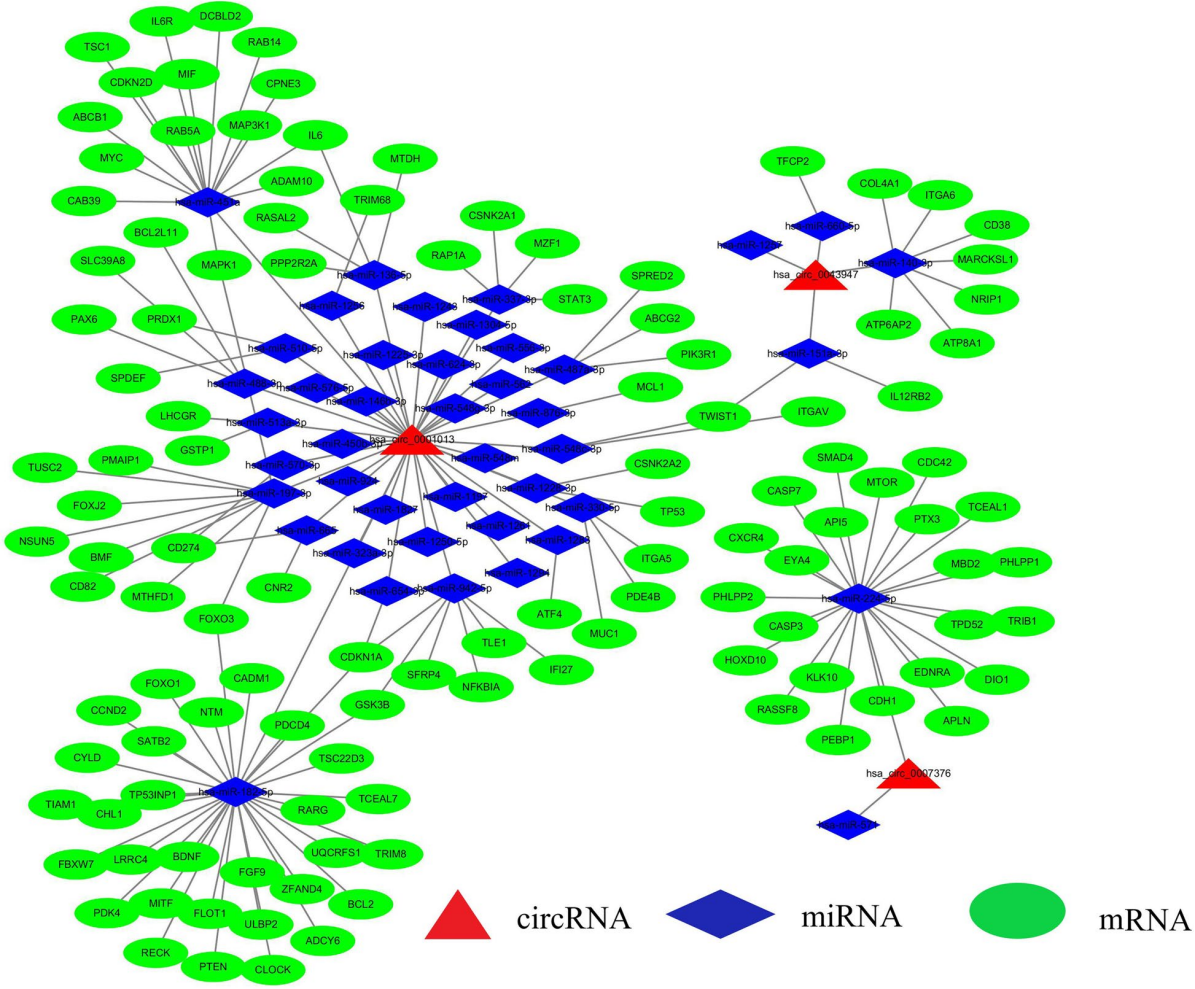
The circRNA-miRNA-mRNA network was constructed based on 3 circRNAs, 43 miRNAs, and 119 mRNAs

We identified 119 experimentally strongly supported target genes of 43 miRNAs by using the mirTarBase online tool (Table 2). Then, we used 3 circRNAs, 43 miRNAs, and 119 mRNAs in Cytoscape 3.6.1 to construct a circRNA-miRNA-mRNA visualization network (Fig. 5).

### Functional and pathway enrichment analysis and PPI network

**Fig. 6:**
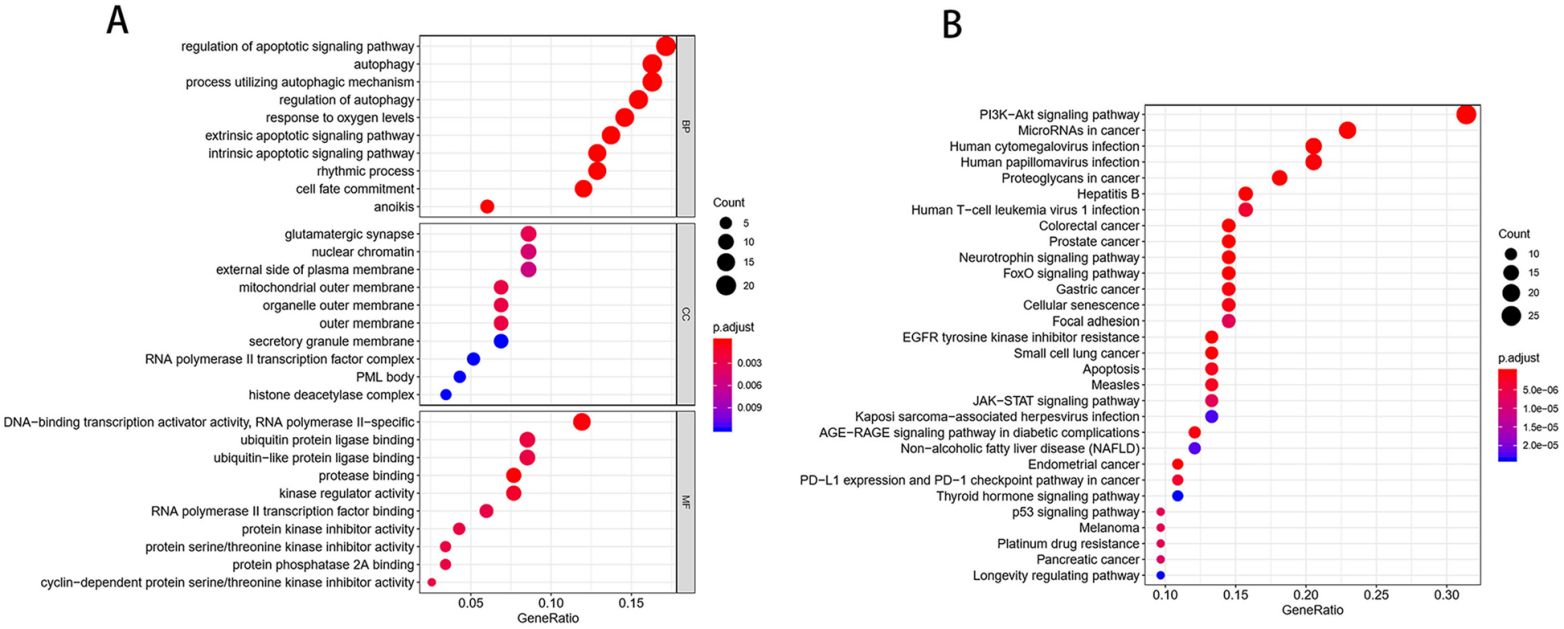
GO and KEEG pathway analysis for 119 mRNAs. **A** Top 10 enriched gene ontology (GO) terms. **B** Top 30 significant KEGG pathways. *KEEG* Kyoto Encyclopedia of Genes and Genomes, *BP* biological process, *CC* cellular component, *MF* molecular function

**Fig. 7:**
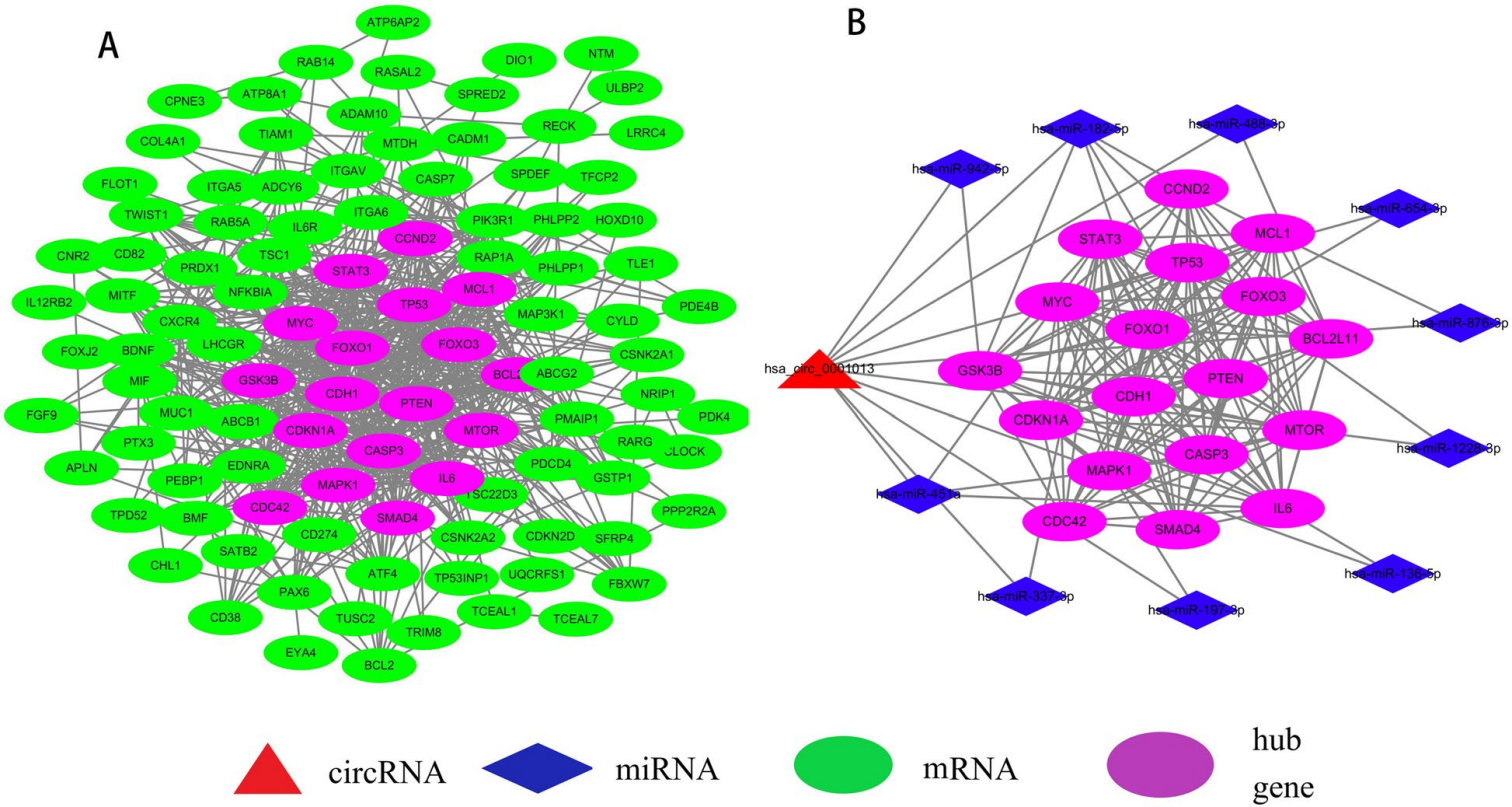
PPI network and core circRNA-miRNA-mRNA network. **A** PPI network composed of 165 nodes and 170 edges, and hub genes identified from the PPI network by Cytoscape. **B** The core circRNA-miRNA-mRNA network based on 1circRNA, 10 miRNAs, and the 18 hub genes. circRNA, circular RNA; miRNA, microRNA; mRNA, messenger RNA; PPI, protein-protein interaction

GO analysis indicated that the 119 mRNAs were mainly enriched in “regulation of apoptotic signaling pathway,” “autophagy,” and “process utilizing autophagic mechanism” (BPs); “glutamatergic synapse,” “nuclear chromatin” and “external side of plasma membrane” (CCs); and “DNA-binding transcription activator activity, RNA polymeraseII-specific” (MFs) (Fig. 6A). KEGG pathway analysis revealed strong enrichment in the “PI3K-Akt signaling pathway” (Fig. 6B). After obtaining the target genes of candidate miRNAs, we created a PPI network composed of 165 nodes and 170 edges (Fig. 7A). Following the identification of the vital functions of hub genes in the network, 18 hub genes (CCND2, STAT3, TP53, MCL1, MYC, FOXO1, FOXO3, BCL2L11, PTEN, MTOR, CDH1, CASP3, IL6, GSK3B, CDKN1A, MAPK1, SMAD4, CDC42) were identified in GC using the MCODE plugin, MCODE_Score = 13.76. These hub genes were predicted target genes for hsa-miR-197-3p, hsa-miR-451a, hsa-miR-136-5p, hsa-miR-337-3p, hsa-miR-654-3p, hsa-miR-182-5p, hsa-miR-1228-3p, hsa-miR-942-5p, hsa-miR-488-3p and hsa-miR-876-3p, and all 10 miRNAs were predicted miRNAs for hsa_circ_0001013. The core circRNA–miRNA–mRNA network based on hub genes is displayed in Fig. 7B.

## Discussion

Emerging evidence indicates that circRNAs are frequently dysregulated in various cancers and may play a vital role in cancer progression. Moreover, the increased stability of circRNAs compared with that of linear RNAs in the serum, makes circRNAs vital biomarkers for cancer diagnosis and prognosis. However, the mechanism of circRNA in cancer progression has not been clearly elucidated. Current evidence demonstrates that circRNAs can target miRNAs, often referred to as “miRNA sponges,” to reduce the level of miRNAs and release their targeting inhibition to mRNAs. These studies have shown that the circRNA-miRNA-mRNA axis can play a role as a wide range of gene expression regulatory networks and can be used as a biomarker for cancer diagnosis and prognosis. Gastric cancer is one of the most common malignant tumors of the digestive tract. At present, radical resection is the main treatment for gastric cancer, but the prognosis of patients is still not satisfactory. Previous studies have confirmed that circRNAs are involved in the tumorigenesis and progression of gastric cancer. Wu et al. investigated the functions of circRNA ring finger protein 111 (circ-RNF111) in GC and found that Circ-RNF111 was higher expressed in GC tissues. Silencing of circ-RNF111 restrained cell viability, colony formation, migration, invasion, cell cycle process, and glycolysis and induced apoptosis in GC cells by regulating KLF12 expression via absorbing miR-876-3p [20]. Liu et al. found that circ-PVT1 contributes to paclitaxel resistance in gastric cancer cells through the regulation of ZEB1 expression by sponging miR-124-3p [21]. Xie et al. showed that the downregulated expression of hsa_circ_0074362 in gastric cancer is related to lymph node metastasis and has diagnostic value for gastric cancer [22]. The expression of hsa_circ_0000190 in gastric cancer tissue and serum is downregulated compared to that in normal samples, suggesting that it may be a more potential biomarker of gastric cancer than the common tumor markers CEA and CA19-9 [23]. Liu et al. attempted to construct the regulatory network of circRNA-miRNA-mRNA in gastric cancer. Their study focused on three downregulated circRNAs (hsa_circ_0001190, hsa_circ_0036287 and hsa_circ_0048607) in gastric cancer tissues and plasma and successfully established the circRNA-miRNA-hub gene network through bioinformatics analysis [24]. However, biomarkers with relatively low abundance are less sensitive to detection than those with high abundance. Therefore, based on previous studies, we attempted to find highly expressed circRNAs in gastric cancer tissues and plasma and to further improve the circRNA-miRNA-mRNA regulatory network to provide a theoretical basis for the study of gastric cancer. In our study, we screened the circRNA expression profiles in the GSE89143 and GSE93541 GEO datasets for gastric cancer tissue and plasma ssample data to identify differentially expressed circRNAs, with the significance threshold set as *P* < 0.05 and |log2FC| > 1.5. Three upregulated circRNAs were selected for further analysis, namely hsa_circ_0001013, hsa_circ_0007376, and hsa_circ_0043947. They have not been reported until now.

Currently, it is generally believed that circRNAs have miRNA response elements (MREs) and can interact with miRNA through a “sponging” action. CiRS-7 was the first circRNA to be reported to act as a ceRNA [25] and circHECTD1 has been shown to act as a ceRNA to promote gastric cancer proliferation by sponging miR-1256 [26]. We also screened 43 miRNAs that may interact with the three selected circRNAs through bioinformatics analyis, and the GO analysis showed that these 43 miRNAs were involved in the regulation of nucleobase, regulation of cell growth, etc. These biological processes are also very active in the development and progression of tumors. We further predicted the downstream target genes of these 43 miRNAs by an online tool and a total of 119 target mRNAs were selected. Next, we analyzed these target genes by using GO and KEGG pathway analysis to gain an understanding of the function of the target genes. The GO analysis showed that the target genes mainly participated in the regulation of apoptotic signaling pathway for BP, glutamatergic synapse for CC and DNA-binding transcription activator activity, RNA polymerase II-specific for MF. The KEGG pathway analysis indicated that the most enriched term was the PI3K-Akt signaling pathway, which is one of the most frequently activated downstream signal transduction pathways in human cancer. The PI3K-Akt signaling pathway plays an important role in regulating cell proliferation, growth, and apoptosis. Peng et al. reported that hsa_circ_0010882 promotes the progression of gastric cancer via regulation of the PI3K/Akt/mTOR signaling pathway [27]. We established a protein-protein interaction (PPI) network consisting of 165 nodes and 170 edges and identified 18 hub genes by using the MCODE plugin in Cytoscape. The 18 hub genes have been reported to be associated with gastric cancer, which are CCND2 [28], STAT3 [29], TP53 [30], MCL1 [31], MYC [32], FOXO1 [33], FOXO3 [34], BCL2L11 [35], GSK3B [36], CDKN1A [37], CDH1 [38], PTEN [39], MTOR [40], MAPK1 [41], CASP3, CDC42 [42], SMAD4 [43], and IL6 [44]. All of them were predicted target genes for hsa-miR-197-3p, hsa-miR-451a, hsa-miR-136-5p, hsa-miR-337-3p, hsa-miR-654-3p, hsa-miR-182-5p, hsa-miR-1228-3p, hsa-miR-942-5p, hsa-miR-488-3p, and hsa-miR-876-3p, and all 10 miRNAs were predicted miRNAs for hsa_circ_0001013. Therefore, a core circRNA-miRNA-mRNA regulatory network was constructed based on 1 circRNA, 10 miRNAs, and 18 hub genes which are called gastric cancer-related genes. Finally, hsa_circ_0001013 was determined to play a key role in the pathogenesis of GC. Although the exact mechanisms of circRNAs in gastric cancer are not clear, our results provide insights into the underlying mechanisms of gastric cancer pathogenesis. The results of this study are based solely on bioinformatics models. This is a pilot study and further studies are needed to verify the biological role of these circRNAs in gastric cancer.

## Conclusions

We obtained circRNA expression profiles in gastric cancer tissue and plasma from the GEO database. Three circRNAs that were upregulated in gastric cancer tissue and plasma compared to normal controls were identified as potential regulators. A core circRNA-miRNA-mRNA network was constructed by using bioinformatics methods. We found that hsa_circ_0001013 may act as a ceRNA and play a critical role in carcinogenesis-related pathways. These findings provide a new pathway for mechanistic studies and offer potential biomarkers for GC. Further studies are needed to examine the role of regulatory modules in GC carcinogenesis.

## Data Availability

We declare that the data and materials in this study will be provided free of charge to scientists for noncommercial purposes.
